# “….it is like a single body being controlled by two heads.”: exploring cognitive, social, and affective engagement in L2 collaborative writing

**DOI:** 10.3389/fpsyg.2026.1849413

**Published:** 2026-07-03

**Authors:** Fatma Kaya

**Affiliations:** English Language Teaching Department, Faculty of Education, Sivas Cumhuriyet University, Sivas, Türkiye

**Keywords:** collaborative writing, engagement in L2 writing, language-related episodes (LREs), pairing methods, second language writing

## Abstract

**Introduction:**

Successful collaborative writing relies on productive interaction and cooperation between learners, which makes engagement a crucial factor in the collaborative process. However, little is known about how learner engagement is manifested across different pairing configurations.

**Methods:**

This study explored undergraduate students' engagement in L2 collaborative writing (CW) by comparing two pedagogically common pairing configurations: student-selected pairs and teacher-assigned high-low proficiency pairs, using both quantitative and qualitative data. Twenty pre-service English teachers—10 categorized as higher proficiency and 10 as lower proficiency—at a state university in Türkiye participated in both pairing conditions within a classroom context. Data were collected through transcripts of collaborative conversations, reflective papers, and participant interviews.

**Results:**

Quantitative findings revealed variations in patterns of interaction and focus of collaborative dialogue across the two pairing configurations. Student-selected pairs, which were predominantly proficiency-homogeneous, tended to demonstrate more collaborative interaction, whereas teacher-assigned high–low pairs engaged more frequently in content-focused talk and fewer mechanics-focused discussions. Although most LRE types and resolved LREs did not differ significantly, student-selected pairs with lower-proficiency core participants showed a higher number of unresolved LREs compared to teacher-assigned high-low pairs. Qualitative findings suggested that student-selected pairings were associated with mutual learning opportunities and emotional comfort, teacher-assigned high-low pairs, however, were more frequently associated with unequal participation and emotional tension particularly for less proficient students.

**Discussion and conclusion:**

Overall, the findings suggest that pairing configuration is linked to variation in different dimensions of learner engagement. Because of this, how pairing is structured in classroom contexts—including decisions regarding selection procedures and proficiency composition—may be an important issue for teachers to address when planning collaborative writing activities.

## 1 Introduction

Collaborative tasks are valuable tools in classrooms that adopt student-centered pedagogies, as interaction within these tasks is likely to enable students to collaboratively evaluate their language use and address any challenges regarding language (Tsoi and Aubrey, 2023). Collaborative writing (CW) aligns with the sociocultural perspective as it enhances the learning process through facilitating mutual support and helping learners reach their potential by engaging in meaningful interaction with peers (Chen, 2019). Research acknowledges that collaborative writing tasks are beneficial for addressing and enhancing L2 writing components including grammar, vocabulary and mechanics (Azkarai and Calzada, 2024; Pan and Teng, 2025).

Recent decades have witnessed a good number of studies investigating learners' collaborative writing performance and its effects on their L2 writing proficiency. More recently, however, CW is increasingly understood as a socially situated process that enables learners to jointly attend to language through interaction with their peers (Zhang, 2025). Given that successful collaborative writing lies in mutual support and meaningful interaction which are central to learning from a sociocultural perspective (Chen, 2019), understanding how learners are engaged in the process is of crucial importance. Despite this, studies examining students' engagement in collaborative work are relatively scarce, particularly in relation to different pairing approaches (Basterrechea and Gallardo-del-Puerto, 2020, 2023; García Mayo and Imaz Aguirre, 2019; Tsoi and Aubrey, 2023; Zabihi and Ghahramanzadeh, 2022). Therefore, this study examines how engagement in collaborative writing is manifested across two naturally occurring pairing types—student-selected pairs and teacher-assigned high–low proficiency pairs—in an undergraduate program.

## 2 Literature review

### 2.1 Learner engagement in L2 collaborative writing

Recent research has examined learner engagement in CW, as higher engagement is a strong predictor of successful performance (Azkarai and Calzada, 2024; Cao et al., 2026; Namkung and Kim, 2024; Paspali, 2025; Wang, 2024; Zabihi and Ghahramanzadeh, 2022; Zhang, 2025). Engagement refers to “a multidimensional construct that includes cognitive, behavioral, social, and emotional dimensions of engagement among second and foreign language learners in the classroom” (Philp and Duchesne, 2016, p. 1). Among the frameworks used to investigate engagement in L2 learning, Engagement with Language (hereafter EWL) provides a useful lens for examining how cognitive, social, and affective factors interact in L2 learning (Svalberg, 2009, 2012). According to Svalberg (2009), EWL describes a cognitive, social, or emotional state, as well as a process where the learner takes an active role, with language as the primary focus. Engagement in collaborative tasks is shaped not only by learners' individual efforts but also by their interactions with peers, making pair dynamics a key consideration (Basterrechea and Gallardo-del-Puerto, 2023; Wang, 2024). Consequently, further research is needed to understand interconnections among cognitive, social and affective dimensions of engagement of L2 learners (Cao et al., 2026).

To examine these dimensions of engagement in CW, the present study adopts Svalberg's (2009, 2012) Engagement with Language (EWL) framework in L2 tasks. Her framework conceptualizes engagement as comprising three interconnected dimensions: cognitive, social, and affective. Cognitive engagement involves a learners' attentiveness, sustained focus and ability to build knowledge through thinking and making logical conclusions. Social engagement is related to learners' preparedness to participate in interaction and assist each other through collaboration. Affective engagement is learners' feelings including joy, willingness and interest in addition to their attitudes and reflections following the completion of a task (Chen et al., 2023; Phung, 2016; Zabihi and Ghahramanzadeh, 2022).

Previous research has demonstrated that learners' engagement may vary according to factors such as proficiency, partner relationships, and interaction patterns. For example, Zabihi and Ghahramanzadeh (2022) found that engagement was low when higher proficiency students were paired with lower proficiency students to complete the writing task. On the other hand, high–high proficiency pairs exhibited higher levels of cognitive and social engagement whereas low–low proficiency pairs displayed increased affective engagement while working on a writing task together. The study conducted by Namkung and Kim (2024) revealed that low proficiency pairs demonstrated better cognitive engagement by focusing more on grammar during video chats, and closeness between partners had a positive influence on social engagement. Moreover, Cao et al. (2026) reported that social mutuality was a crucial factor in facilitating or hindering engagement, and at the individual level, learners' engagement profiles were shaped by their perceived social positioning and contributions within the group. In another study by Azkarai and Calzada (2024), young learners were found to display higher levels of social and cognitive engagement. Moreover, their affective engagement was higher when they adopted a collaborative interaction pattern. However, they tended to avoid providing explicit support or encouragement. Lastly, Zhang (2025) found that social and affective experiences influenced learners' engagement in collaborative writing. Learners who interacted actively and perceived the task positively exhibited higher levels of cognitive engagement, whereas socially disengaged learners showed less attention to addressing language-related problems. Taken together, these findings suggest that engagement in collaborative writing is influenced by factors such as proficiency pairing, partner relationships, and interaction patterns.

### 2.2 Collaborative writing and pairing methods

Research on collaborative writing (hereafter CW) has revealed that learners can benefit from CW to improve their L2 writing skills including fluency, accuracy and organization (Abdeta et al., 2026; Chen, 2019; Bikowski and Vithanage, 2016; Dobao, 2012; McDonough et al., 2018; Pan and Teng, 2025; Wang et al., 2025). A substantial body of research has focused on language-related episodes (hereafter LREs) produced by the learners when they work on a writing task collaboratively (Basterrechea and Gallardo-del-Puerto, 2020). According to Swain and Lapkin (1998), LREs refer to moments during collaborative talk where learners reflect on how they use the language, monitor it or attempt for self/peer correction. Moreover, patterns of interaction have been extensively explored in the context of CW (Chen, 2019), with much of this work being based on Storch's model. Storch (2002) identified four interaction patterns: collaborative, dominant/dominant, dominant/passive, and expert/novice, noting that collaborative and expert/novice patterns tend to yield positive learning outcomes.

In addition to interaction patterns and LREs, researchers have explored how different pairing methods shape learners' collaborative experiences and outcomes. Studies comparing student-selected pairs with teacher-assigned pairs found that learners tended to be more satisfied with self-selected pairing, as they worked in a friendlier atmosphere and enjoyed collaboration more when they chose their own partners (Tsoi and Aubrey, 2023). In their study, Liu et al. (2025) concluded that familiar pairs generated significantly more LREs and resolved a greater proportion of them compared to unfamiliar pairs. On the other hand, several studies revealed that student-selected pairs led to negative outcomes including off-task talks, an inability to solve language problems (García Mayo and Imaz Aguirre, 2019; Mozaffari, 2017). Basterrechea and Gallardo-del-Puerto (2023) explored whether there was a difference between student-selected and proficiency matched groups regarding their performance in a L2 writing task. Their study revealed that proficiency matched groups outperformed student selected groups in generating LREs that closely approximated the target equivalence. Similarly, Mozaffari (2017) found that teacher assigned pairs were more successful in terms of fluency and accuracy than student-selected pairs. These findings indicate that pairing configuration influences both the quality of interaction and the resolution of language-related problems.

Another strand of studies focused on pairing students based on their proficiency level. Watanabe and Swain (2007) explored four core participants who worked with higher proficiency students and lower proficiency students. Their study demonstrated that differences in proficiency did not have a significant influence on collaboration and L2 performance. In contrast, Wang et al. (2026) found that proficiency grouping influenced writing outcomes, as mixed-proficiency dyads produced texts with significantly lower linguistic complexity than same proficiency dyads. In their study, Niu et al. (2018) found that low–low pairs displayed a better performance in producing LREs and employing scaffolding strategies compared to high–high and high–low pairs. However, high–high and high–low pairs were more successful in resolving LREs compared to low–low pairs. Nevertheless, Storch and Aldosari (2013) reported that high–low pairs produced fewer LREs especially when their pattern of interaction was dominant passive in their study. Moreover, high–high pairs concentrated more on language use while low–low pairs were more interested in formulating ideas for the essay.

Overall, while existing research indicates that pairing configurations influence collaborative writing processes, there is still no clear agreement on which pairing types are most effective and under what conditions (Li and Zhang, 2021). Studies have examined different pairing variables, such as partner self-selection, peer familiarity, and proficiency composition (Basterrechea and Gallardo-del-Puerto, 2023; Liu et al., 2025; Niu et al., 2018; Tsoi and Aubrey, 2023; Wang et al., 2026), and have assessed collaborative writing using different outcome measures, including LREs, interaction patterns, learner engagement, and text quality. As a result, pairing arrangements that seem beneficial based on one measure may not necessarily be regarded as effective according to another. In addition, within-subjects investigations of engagement across different classroom pairing configurations remain underexplored (Tsoi and Aubrey, 2023), as most previous studies have used between-subjects designs (Basterrechea and Gallardo-del-Puerto, 2020, 2023; García Mayo and Imaz Aguirre, 2019; Zabihi and Ghahramanzadeh, 2022).

The present study employs a within-subjects design to examine engagement across student-selected and teacher-assigned high–low proficiency pairings among Turkish pre-service English teachers, using both interactional and self-report data. Given the importance of engagement for successful collaboration, further research is needed to examine how learners' engagement is manifested across different pairing configurations. Accordingly, the present study adopts a multidimensional view of engagement, focusing on its cognitive, social, and affective dimensions.

Following Zabihi and Ghahramanzadeh (2022) and Azkarai and Calzada (2024), this study examined learner engagement in collaborative writing at both the pair and individual levels. This distinction was adopted because engagement in collaborative tasks is manifested both through learners' subjective experiences and their interactional behavior. At the individual level, engagement was explored through learners' reflections and interviews, which provided evidence of their cognitive, social, and affective engagement. At the pair level, engagement was examined through recordings of collaborative interaction. Drawing on Svalberg's (2009, 2012) framework, social engagement was operationalized through patterns of interaction that demonstrate the extent of learners' mutual participation, negotiation, and support during collaboration, whereas cognitive engagement was captured through language-related episodes (LREs), which reflect learners' attention to language, problem solving, and joint knowledge construction in addition to discussions related to content and organization. Similar interactional measures have been used in previous collaborative writing and engagement research to examine how engagement is enacted during task-based interaction (e.g., Azkarai and Calzada, 2024; Zabihi and Ghahramanzadeh, 2022; Zhang, 2025). Thus, pair-level engagement in the present study refers to the interactional manifestation of engagement (cognitive and social) during collaborative writing, whereas individual-level engagement refers to learners' self-reported cognitive, social, and affective experiences of the task. Because affective engagement is not directly observable in interactional data, it was examined only through learners' reflections and interview accounts. To this end, the study seeks to answer the following research questions:

- How do student-selected pairs and teacher-assigned high–low proficiency pairs differ in the interactional manifestations of cognitive and social engagement during collaborative writing?
- How do learners in student-selected pairs and teacher-assigned (high–low proficiency) pairs differ in their perceived cognitive, social, and affective engagement during collaborative writing?

## 3 Method

### 3.1 Context and participants

The study was carried out at a state university in Sivas, Türkiye. The participants were undergraduate students who were pre-service English teachers. They were freshman students who were required to take basic courses related to English including language skills (speaking, listening, reading and writing) and grammar. The study was conducted in L2 writing class where they were expected to write their essays in pairs. The core participants comprised 20 pre-service English teachers (12 female and 8 male), aged between 18 and 20 years, including 10 higher-proficiency and 10 lower-proficiency students. The participants with higher proficiency were assigned the codes P1 through P10, while participants with lower proficiency were labeled as P11 through P20.

### 3.2 Data collection tools and procedure

The researcher adopted case study research design and used both quantitative and qualitative data collection tools including participants' collaborative talk records, reflection papers and semi-structured interviews with the participants. Purposive sampling was adopted in the present study as language proficiency of the students was one of the crucial variables of the study. Proficiency levels were determined using their fall term writing class scores as this study was conducted during the spring term. Students whose average scores ranged between 80 and 100 were categorized as more proficient, whereas those with scores between 40 and 60 were considered as less proficient. These thresholds were selected based on institutional grading norms, where scores above 80 typically indicate high achievement and scores between 40 and 60 reflect a basic but incomplete performance. The researcher was also the instructor of the L2 writing course in which the study was conducted. Participation in the study was voluntary, and students were informed that their decision to participate or not participate would have no effect on their course grades. Written informed consent was obtained from all participants prior to data collection. To reduce potential researcher bias, a second researcher independently coded the qualitative and interactional data, and coding discrepancies were discussed until agreement was reached.

The study lasted for 7 weeks and involved 30 participants in total for collaborative talk recordings, forming interactional dyads. However, the qualitative analysis focused on a core group of 20 participants (P1–P20), who took part in both pairing conditions and constituted the within-subjects sample. Participants P21–P30 were included only in interactional dyads during the collaborative talk phase (only in student-selected session) and were not part of the repeated-measures qualitative comparison.

First, in order for students to get familiarized with collaborative writing, they were asked to write an essay (cause-effect) with a pair selected by the teacher (random pairing) and got feedback related to their writing performance from the teacher. Next, for the session where they chose their pairs, participants with mixed proficiency levels wrote a definition essay collaboratively and recorded their discussions. In the student-selected pairs, proficiency configurations inherently varied. Among pairs with higher-proficiency core participants, six pairs were high–high and four were high–low. Among pairs with lower-proficiency core participants, eight pairs were low–low and two were high–low. Hence, student-selected condition included mainly pairs with similar proficiency, whereas mixed-proficiency pairings were also observed. In the following week, they were asked to revise their essay collaboratively and asked to reflect on their experiences individually. For this purpose, students were asked to answer predefined open-ended questions. These questions included: “How did you find the experience of writing a composition with a friend?”, “What was the most challenging aspect for you?”, “What did you enjoy most in this process?”, and “What are the advantages/disadvantages of writing together?” Questions were adapted from Shehadeh (2011) and Chen (2021).

Subsequently, the same process was followed with the teacher assigned pairs, in which they wrote a comparison-contrast essay. For teacher-assigned pairs, the instructor chose 10 successful and 10 less successful students among 90 students who participated in the previous teacher assigned (random) and student-selected sessions. While deciding on pairs, the teacher paired successful students with less successful ones, and they constituted core participants of the study. By contrast, teacher-assigned pairs consisted of deliberately arranged high–low pairs.

Finally, semi-structured interviews were conducted with core participants (*n*: 20) to allow them to clarify their statements in their reflective papers and present deeper insights into their perceptions related to collaborative writing with different pairings. Interviews were audio-recorded and each interview session lasted for approximately 30 min Different essay genres (definition and comparison–contrast) were used across pairing conditions in order to reduce potential task repetition effects. Moreover, participants had prior exposure to multiple genres in curriculum.

A within-subjects design was employed for the core group of 20 participants (10 higher-proficiency and 10 lower-proficiency students), all of whom took part in two collaborative writing sessions. In Session 1, each participant worked with a self-selected partner, who was not included in Session 2. In Session 2, the same 20 participants were re-paired by the researcher, typically matching higher- and lower-proficiency students together. This design allowed for a comparison of the core participants' engagement across two naturally occurring classroom pairing configurations. While the first pairing configuration reflected students' natural selection preferences, the second was pedagogically structured in line with Lev Vygotsky's Sociocultural Theory (1978), particularly the concept of the Zone of Proximal Development (ZPD). According to this framework, learners improve more effectively when they work with a more knowledgeable peer who can scaffold their performance. In this study, higher-proficiency students were expected to function as “more knowledgeable others,” supporting their less-proficient partners during the collaborative writing tasks. The timeframe for the study was illustrated in Table 1.

**Table 1:** Timeframe for data collection procedure.

Week	Learning activities
1	Collaborative writing (for familiarization)
2	Revision on collaboratively written essay
3	Collaborative writing (with sts-selected pairs)
4	Revision on collaboratively written essay (with sts-selected pairs)-writing reflection related to their experience
5	Collaborative writing (with teacher assigned pairs)
6	Revision on collaboratively written (with teacher assigned pairs)-writing reflection related to their experience
7	Follow-up interview with participants on their experiences related to collaborative writing

### 3.3 Data analysis

Collaborative talk records (*n*: 30), reflection papers written twice by the participants and semi-structured interviews with the participants were analyzed by the author. The analytical procedures were structured according to the two research questions. RQ1, which focused on the interactional manifestations of cognitive and social engagement, was addressed through the analysis of collaborative talk recordings. Cognitive engagement was examined through language-related episodes (LREs) as well as content- and organization-related discussions, whereas social engagement was examined through patterns of interaction. Frequencies and percentages were calculated and compared across pairing configurations using descriptive statistics and Wilcoxon tests. RQ2, which focused on learners' perceived engagement, was addressed through reflection papers and semi-structured interviews. These qualitative data were analyzed using content analysis and coded according to Svalberg's (2009, 2012) dimensions of cognitive, social, and affective engagement. The qualitative findings were then compared across the two pairing configurations to identify similarities and differences in learners' engagement experiences.

Following Zabihi and Ghahramanzadeh (2022) and Azkarai and Calzada (2024), this study investigated learner engagement in collaborative writing at both the pair and individual levels. Reflections and interviews were analyzed to examine cognitive, affective, and social engagement at the individual level, while recordings of collaborative talk were analyzed to explore interactional manifestations of cognitive (i.e., language-related episodes) and social (i.e., patterns of interaction) engagement at the pair level. Affective engagement at the pair level was not explored, as positive emotions (e.g., laughter) were limited and negative emotions (e.g., disappointment) were difficult to detect in the recordings. Collaborative talk records (*n* = 30) included three different groups: one with high-proficiency level students (*n* = 10) and their selected pairs, one with lower-proficiency level students (*n* = 10) and their selected pairs (these two groups were involved in collaborative writing in the same session-student selected pairs), and the last one with high-proficiency level students and lower-proficiency level students paired (*n* = 10). For analyzing collaborative talk records, coding procedures for patterns of interaction and language related episodes were adopted following Storch (2002) and Swain and Lapkin (2001), respectively by the researcher. However, the frequency of talk related to content and organization of the essay was also considered in addition to language related episodes, as these were commonly observed in collaborative talk records and are integral to essay writing performance. A detailed coding approach was used. The transcribed collaborative writing interactions were initially divided into turns. Each turn was then coded individually for interaction patterns (collaborative, dominant/passive, dominant/dominant, expert/novice), as well as for LRE type (form-, lexis-, or mechanics-focused), and for content- and organization-related talk. After coding each individual instance, all occurrences were counted for each dyad. These counts were then converted into percentages to allow comparison across pairs. In this way, the analysis focused on small interactional units rather than assigning a single overall interaction pattern to each dyad (see Supplementary Material for illustrative transcripts).

Coding was repeated by a colleague with 12 years of teaching experience and a Ph.D. in the relevant field. As a result, 90 % of agreement was found between researchers. Moreover, Jamovi version 2.6.26 was used to analyze the quantitative data derived from the collaborative talk records. Both descriptive and inferential statistics were conducted. Since the data were not normally distributed and the sample size was small, Wilcoxon tests—a non-parametric method—were used to investigate whether there were any significant differences between teacher-assigned pairs and student-selected pairs with core participant with high proficiency level, and between teacher-assigned pairs and student-selected pairs with core participant with low proficiency level (within groups) in terms of patterns of interaction and language-related episodes employed by the pairs.

For analyzing qualitative data including reflective papers and semi-structured interviews with the participants, the researcher employed content analysis. The qualitative data analysis involved an iterative and systematic procedure applied to the entire dataset, including reflection papers and interview transcripts from both pairing configurations. Following an inductive approach, the researcher adopted the steps of creating codes, categorizing them and interpreting them (Lindgren et al., 2020). Firstly, the data were read by the researcher reiteratively for thorough understanding of it (Elo and Kyngas, 2008). Second, relevant and recurring statements across the full dataset were highlighted for initial coding. Third, these codes were compared within and across pairing conditions in an iterative process to uncover recurring patterns and variations, and codes that did not align with the research focus were removed. Fourth, the codes were refined and grouped into broader categories. To gain an in depth understanding of how participants were engaged in group work, a multi-dimensional perspective was adopted, and categories were compared and grouped in accordance with cognitive, social and affective dimensions (Svalberg, 2009, 2012). This process led to the development of a single, unified coding framework that was applied consistently to all participants. Although the same coding framework was used for the entire dataset, certain codes and categories were more prominent in one pairing condition than in the other. Therefore, separate tables were used to present and compare these patterns more clearly.

Several categories were grouped under more than one dimensions as they were related to more than one dimension (see Tables 2, 3). During the qualitative data collection process, participants first language was used to eliminate potential misunderstandings and ambiguity which may arise because of participants' limited proficiency in L2. To ensure the reliability of the qualitative data analysis process, the researcher invited a colleague with 12 years of teaching experience and a Ph.D. in the relevant field to analyze the qualitative data. As a result of discussing the coding process for the reflective papers and the interview data, a 90 % of agreement was found between researchers. To enhance validity, the researcher triangulated the data through using a variety of data collection tools including collaborative talk records, reflective papers and semi-structured interviews.

**Table 2:** Codes and categories of the qualitative data for sts-selected pairs with mixed-proficiency.

Categories	Codes
1. Cognitive dimension	
1.1. Self-improvement through collaboration	Noticing own weaknesses through peer writing
	Peer-assisted L2 learning
	Peer correction
2. Affective dimension	
2.1. Emotional comfort/discomfort	Expressing ideas freely and comfortably
	Feeling less successful than the partner
2.2. Interpersonal relationship dynamics	Tolerance of opposing views
	Fear of hurting friend (accepting ideas)
	Mutual support and effort
3. Social dimension	
3.1. Collaboration for task completion	Exchanging ideas about essay topic
	Creating a common piece (writing) from different thoughts
	Reaching an agreement despite differences of opinion
3.2. Support for L2 writing skills improvement	Peer correction
	Peer-assisted L2 learning
3.3. Challenges in collaboration	Difficulty of coordination
	Difficulty of finding common ground
	Waste of time in negotiation

**Table 3:** Codes and categories of the qualitative data for teacher-assigned high–low pairs.

Categories	Codes
1. Cognitive dimension	
1.1. Self-improvement through collaboration	Learning new phrases/vocabulary from the pair
	Producing a more successful essay
1.2. Cognitive burden in strategic collaboration	taking more responsibility while writing
	lack of mutual benefit
1.3. Focus on production	Being more task oriented
2. Affective dimension	
2.1. Emotional comfort/discomfort	Hesitating to express ideas
	Ideas rejected by partner
2.2. Interpersonal relationship dynamics	Having to take more responsibility while writing
	Lack of mutual benefit
3. Social dimension	
3.1. Collaboration for task completion	Exchanging ideas about essay topic
	Creating a common piece (writing) from different thoughts
3.2. Support for L2 writing skills improvement	Peer assisted L2 learning
	Learning about L2 writing from the peer
3.3. Challenges in collaboration	Difficulty of finding common ground
	Waste of time in negotiation
	Convincing the partner of errors
	Writing being more suitable to do individually

## 4 Findings

This case study explored how English language learners participated in collaborative writing, both in student-selected pairs and teacher-assigned pairs. The following sections present statistical findings regarding the pair level manifestations of learners' social and cognitive engagement, as reflected in patterns of interaction and language related episodes in addition to content and organization related talks across two collaborative writing sessions (one student-selected and one teacher-assigned), followed by qualitative findings concerning learners' cognitive, social, and affective experiences of engagement based on their reflections and interview accounts.

### 4.1 Pair-level manifestations of engagement: patterns of interaction and language-related episodes

Table 4 demonstrates percentages and frequencies of patterns of interaction in student-selected pairs in which core participants (one of the peers in each pair) are with higher proficiency level. As shown in the table below, collaborative pattern (with 69%) was the most frequently observed patterns of interaction in student-selected pairs. It was followed by expert/novice (with 15%) and dominant/dominant (with 10%) patterns of interaction respectively. Dominant/passive (with 6%) pattern was found to be the least frequently employed patterns of interaction.

**Table 4:** Patterns of interaction in student-selected pairs (core participants with higher proficiency level).

Pairs	Collaborative	Dominant/passive	Dominant/dominant	Expert/novice
Pair 1	67% (10)	13% (2)	7% (1)	13% (2)
Pair 2	91% (10)	9% (1)	–	–
Pair 3	85% (17)	5% (1)	–	10% (2)
Pair 4	81% (17)	5% (1)	5% (1)	9% (2)
Pair 5	68% (17)	8% (2)	12% (3)	12% (3)
Pair 6	47% (9)	16% (3)	–	37% (7)
Pair 7	57% (8)	–	–	43% (6)
Pair 8	68% (21)	6% (2)	23% (7)	3% (1)
Pair 9	67% (14)	–	14% (3)	19% (4)
Pair 10	57% (8)	–	29% (4)	14% (2)
Total	69% (131)	6% (12)	10% (19)	15% (29)

As highlighted in Table 5, collaborative pattern (with 66%) was the major pattern of interaction adopted by the participants in student-selected pairs in which core participants (one of the peers in each pair) were with lower proficiency level. While dominant/dominant (with 15%) and dominant/passive (14%) interaction patterns followed it, expert/novice pattern (5%) was rarely observed.

**Table 5:** Patterns of interaction in student-selected pairs (core participants with lower proficiency level).

Pairs	Collaborative	Dominant/passive	Dominant/dominant	Expert/novice
Pair 1	59% (10)	12% (2)	12% (2)	17% (3)
Pair 2	67% (12)	33% (6)	–	–
Pair 3	60% (12)	30% (6)	10% (2)	–
Pair 4	50% (11)	–	41% (9)	9% (2)
Pair 5	53% (10)	21% (4)	10% (2)	16% (3)
Pair 6	75% (15)	25% (5)	–	–
Pair 7	79% (23)	–	17% (5)	4% (1)
Pair 8	68% (21)	6% (2)	23% (7)	3% (1)
Pair 9	75% (9)	–	17% (2)	8% (1)
Pair 10	72% (23)	16% (5)	12% (4)	–
Total	66% (146)	14% (30)	15% (33)	5% (11)

Collaborative pattern (with 45%) was employed slightly more frequently than the dominant/passive pattern (with 44%) by the participants in teacher assigned pairs, where successful students were paired with less successful students as indicated in Table 6. Expert/novice pattern (with 11%) was also adopted by the participants whereas dominant/dominant pattern was not observed.

**Table 6:** Patterns of interaction in teacher-assigned pairs.

Pairs	Collaborative	Dominant/ passive	Expert/ novice
Pair 1	69% (11)	31% (5)	–
Pair 2	19% (4)	52% (11)	29% (6)
Pair 3	56% (10)	39% (7)	5% (1)
Pair 4	50% (9)	39% (7)	11% (2)
Pair 5	43% (9)	38% (8)	19% (4)
Pair 6	62% (10)	19% (3)	19% (3)
Pair 7	48% (9)	47% (9)	5% (1)
Pair 8	53% (9)	35% (6)	12% (2)
Pair 9	35% (7)	60% (12)	5% (1)
Pair 10	25% (4)	75% (12)	–
Total	45% (82)	44% (80)	11% (20)

Inferential statistics were run to investigate whether there were significant differences in patterns of interaction across student-selected and teacher-assigned pairing configurations. Wilcoxon tests revealed that there was a significant difference between student-selected pairs with core participant with high proficiency level and teacher assigned high–low pairs in terms of collaborative and dominant-passive interaction patterns with *p* < 0.05: collaborative: *p* = 0.032, *r* = −0.782; dominant-passive: *p* = 0.009, *r* = 1.000. However, there was not statistically significant difference between groups in terms of expert-novice interaction pattern (*p* = 0.284, *r* = −0.422). Wilcoxon tests further showed that there was a significant difference between student-selected pairs with core participant with low proficiency level and teacher assigned high–low pairs in terms of collaborative and dominant-passive interaction patterns with *p* < 0.05: collaborative: *p* = 0.009, *r* = −0.945; dominant-passive: *p* = 0.011, *r* = 0.927. There was not statistically significant difference between groups in terms of expert-novice interaction pattern (*p* = 0.242, *r* = −0.571).

Table 7 shows that group 1(teacher assigned) generated 69 (*M* = 6.90) form-focused LREs, 59 (*M* = 5.90) lexis-focused LREs, and 6 (*M* = 0.600) mechanics-focused LREs. Moreover, content-related issues were discussed 164 (*M* = 16.4) times, and organization-related talks were produced 20 (*M* = 2.00) times. On the other hand, group 2 (sts with higher proficiency-selected) produced 63 (*M* = 6.30) form-focused LREs, 72 (*M* = 7.20) lexis-focused, and 24 (*M* = 2.40) mechanics-focused LREs. While content-related talks were observed 79 (*M* = 7.90) times, 19 (*M* = 1.90) organization-related talks were produced. Lastly, 76 (*M* = 7.60) form-focused LREs, 66 (*M* = 6.60) lexis-focused LREs, and 33 (*M* = 3.30) mechanics-focused LREs were produced by pairs in group 3 (sts with lower proficiency-selected) in addition to 73 (*M* = 7.30) content-related talks, and 30 (*M* = 3.00) organization-related talks.

**Table 7:** Language-related episodes and content- and organization-related talk across the three groups.

		Group 1 (teacher assigned)		Group 2 (sts with higher proficiency-selected)		Group 3 (sts with lower proficiency-selected)	
Classification	Category	*n*	*M* (*SD*)	*n*	*M* (*SD*)	*n*	*M* (*SD*)
Focus	Form-focused	69	6.90 (2.56)	63	6.30 (3.62)	76	7.60 (2.84)
	Lexis-focused	59	5.90 (3.35)	72	7.20 (4.18)	66	6.60 (2.84)
	Mechanics-focused	6	0.600 (0.69)	24	2.40 (1.71)	33	3.30 (2.63)
	Content	164	16.4 (4.72)	79	7.90 (4.20)	73	7.30 (3.59)
	Organization	20	2.00 (1.05)	19	1.90 (1.52)	30	3.00 (1.15)
Quality of LREs	Correctly resolved	106	10.60 (3.20)	128	12.80 (4.80)	121	12.10 (3.35)
	Incorrectly resolved/unsolved	28	2.80 (1.55)	31	3.10 (0.99)	54	5.40 (2.88)

According to the table, the most frequently correctly resolved LREs were generated by pairs in group 2 (sts with higher proficiency-selected) with a frequency of 128 (*M* = 12.80), it was followed by 121 (*M* = 12.10) correctly resolved LREs by pairs in group 3 (sts with lower-proficiency selected), and 106 (*M* = 10.60) correctly resolved LREs by pairs in group 1(teacher assigned), respectively. The least frequently incorrectly resolved /unsolved LREs were observed in pairs in group 1 (teacher assigned) with a frequency of 28 (*M* = 2.80), and the most frequently incorrectly resolved /unsolved LREs were observed in pairs in group 3 (sts with lower proficiency selected) with a frequency of 54 (*M* = 5.40).

In addition, Wilcoxon tests were run to explore whether there was any significant difference between teacher-assigned and student selected pairs with regard to LREs, content and organization focused talks generated by the pairs and quality of LREs. No significant differences were found for form-focused (*p* = 0.726, *r* = 0.167), lexis-focused (*p* = 0.440, *r* = −0.311), or organization-focused episodes (*p* = 0.746, *r* = 0.190). However, significant differences were observed for mechanics-focused episodes (*p* = 0.022, *r* = −0.867) and content-focused episodes (*p* = 0.015, *r* = 0.933) between teacher assigned pairs and student selected pairs with core participants with high proficiency level, indicating that pairs in the teacher-assigned high–low condition engaged in significantly more content-focused discussions and fewer mechanics-focused discussions compared to pairs in the student-selected condition. Similarly, there were not any statistically significant differences in form-focused (*p* = 0.672, *r* = −0.194), lexis-focused (*p* = 0.918, *r* = −0.054), or organization-focused episodes (*p* = 0.103, *r* = −0.714) between teacher assigned pairs and student selected pairs with core participants with low proficiency level. However, mechanics-focused episodes (*p* =0.028, *r* = −0.888) and content-focused episodes (*p* = 0.006, *r* = 0.927) showed significant differences, revealing that students in teacher assigned high–low pairs generated significantly more content focused discussions and fewer mechanics-focused discussions compared to those in students selected pairs. Moreover, the results indicated that there was no statistically significant difference in the number of solved LREs (*p* = 0.201, *r* = −0.473) and in the number of unsolved LREs (*p* = 0.752, *r* = −0.190) between teacher assigned pairs and student selected participants with core participant with high proficiency level. Moreover, Wilcoxon test did not show any statistically significant difference in the number of solved LREs (*p* = 0.444, *r* = −0.291) between teacher assigned pairs and student selected pairs with core participant with low proficiency level. However, a significant difference was observed in the number of unsolved or incorrectly resolved LREs (*p* = 0.031, *r* = −0.782) between teacher-assigned pairs and student-selected pairs with low-proficiency core participants.

### 4.2 Individual-level experiences of engagement in collaborative writing

Qualitative findings are presented in this section to shed light on participants' cognitive, affective and social engagement at individual level in collaborative writing.

#### 4.2.1 Engagement related to collaborative writing in the student-selected pairing configuration

Twenty core participants (ten successful and 10 less successful) reflected on their collaborative writing experience with a peer whom they chose. And they were interviewed after the study. The results for the cognitive dimension are presented first. According to the participants, they had the opportunity to improve their L2 writing skills through collaboration. They could see the other party's writing strategies and notice their own shortcomings (*n* = 5) as stated by participant 16:

“Before starting to write my partner suggested finding a model essay and analyzing its thesis and topic sentences, then we created our own thesis and topic sentences and compared them with those of the model essay. This process was helpful and made it easier for us to organize our essay.”

In addition, the participants (*n* = 10) learnt from each other about L2 usage and corrected each other's mistakes (*n* = 8). Participant 15 indicated that he learned about complex sentence construction from his peer during collaborative writing, while P19 reported that they noticed and corrected each other's mistakes related to issues such as complex sentence construction and tense agreement.

Regarding the affective dimension, participants indicated both emotional comfort and discomfort. They were able to express their ideas freely and comfortably as they were working with a friend (*n* = 14). Participant 12 claimed that she felt comfortable and expressed her thoughts with no hesitation. Even, they laughed at each other's mistakes and had fun while writing. Another participant (P18) stated that he was not afraid of making mistakes. Since the person he was writing with was his friend, he had no worry that his friend would think he sounded silly if he made a mistake. On the other hand, several participants (*n* = 4) indicated that they experienced a sense of being less successful than their partner as illustrated in comments by P11:

“While writing the essay, I realized that I was using simpler expressions compared to my partner, and I felt discouraged about it.”

Another aspect of affective dimension was interpersonal relationship dynamics in classwork as working with a friend influenced the collaborative writing process in a way. Several participants (*n* = 5) were able to be tolerant of opposing viewpoints. P6 stated that there were disagreements and opposing ideas, but since they were friends, they did not dwell on them and just let it go. There were also mutual support and effort according to the participants (*n* = 6) as displayed in comments by P1 and P6:

“The peer I chose was experienced and knowledgeable. We complemented each other more easily. For example, I had difficulty writing body paragraphs, and my friend supported me. On the other hand, I was better at writing the introduction, so I took more responsibility in that part” (P1).

“While planning from the beginning, we motivated ourselves to create something good together and did our best to achieve it.” (P6)

Nevertheless, several participants (*n* = 4) asserted that they had to accept their friends' ideas out of fear of hurting them. P20 stated that while discussing the content topic, her peer suggested ideas that were not directly relevant to the subject, and she had to approve them in order not to hurt her peers' feelings.

Concerning the social dimension, the participants collaborated for task completion. To achieve this, a significant number of participants (*n* = 15) reported exchanging ideas with the pair related to the essay topic. According to P8, it was rewarding to share her ideas with the peer and research them together as it encouraged open dialogue and collective understanding. Moreover, they (*n* = 7) created a common piece from different thoughts as reflected in comments by P3:

“It was nice to ask each other for our ideas on the topic as we combined them together and created something collectively. Moreover, it was fun to hear ideas from my friend that would never have crossed my mind, and it broadened my horizons.”

Lastly, they (*n* = 4) were able to reach an agreement despite differences of opinion for the sake of completing the task. Participant 17 stated that it was challenging but still, they were able to find a common ground by presenting their reasons to each other and making compromises although they had different perspectives to complete the task.

Collaborative writing contributed to L2 writing skills improvement through peer-learning (*n* = 10) and peer correction (*n* = 8). However, the participants also experienced challenges while writing collaboratively. Being coordinated was difficult according to several participants (*n* = 4). Finding a common ground was also not that easy (*n* = 7) and it led to a waste of time (*n* = 4). Participant 15 said:

“Having contrasting opinions and both sides believing they were right made things challenging. After a while, the discussion reached a deadlock and prevented further progress. It ultimately led to a waste of time.”

#### 4.2.2 Engagement related to collaborative writing in the teacher-assigned high–low pairing configuration

Twenty core participants (ten high-proficiency level and 10 low-proficiency level) reflected on their collaborative writing experience with teacher selected pair. High proficiency level students (*n* = 10) were paired with lower-proficiency level students (*n* = 10) by the teacher, which included core participants (*n* = 20) of this study. And they were interviewed after the study. The results for the cognitive dimension revealed that the participants experienced self-improvement through collaboration. Six participants reported learning new vocabulary or phrases from the peer. Several participants (*n* = 4) claimed that they were able to produce a more successful essay thanks to collaboration as P14 said:

“We were able to generate more ideas and negotiate on essay organization; as a result, we crafted a more successful essay.”

Nevertheless, collaborative writing with a teacher selected pair posed a cognitive burden for the participants. Some participants (*n* = 9) stated that they had to take more responsibility during the collaborative writing process as illustrated in comments by P1 and P4:

“…her ideas and sentences were too simple, and her language proficiency was low. As a result, I had to assist more in the writing process, which I found disturbing.” (P1)

“My peer was not good at essay organization and did not know much about the essay genre. Therefore, I had to explain them to my peer and contribute more to essay writing.” (P4)

In connection with this, mutual benefit was absent according to some participants (*n* = 8) as stated by P5:

“In collaborative writing with a student-selected pair, we had the chance to correct each other's L2 grammar mistakes. However, in collaborative writing with a teacher-selected pair, I was the one who had to correct my peer. If my pair had been knowledgeable, we could have learned from each other, making the experience more beneficial for both of us.”

Finally, several participants (*n* = 5) asserted that they focused on the final production. P9 said:

“Since we were not friends, the atmosphere was tense and serious, and our main goal was to complete the writing task in the best way possible.”

Regarding the affective dimension, emotional discomfort was experienced by the participants. Several participants (*n* = 10) reported hesitating to express their ideas. However, the reasons varied depending on their proficiency level. High-proficiency participants (*n* = 4) hesitated to avoid sounding dominant as illustrated in comments by P7:

“It felt like I was doing most of the writing, so I occasionally stepped back to give my partner an opportunity. I was concerned that taking on too much of the work might make her uncomfortable.” (P7)

On the other hand, low-proficiency participants (*n* = 6) hesitated due to feelings of inferiority and the dominance of the peer. P14 and P17 said:

“I felt inadequate; my peer was presenting great ideas and was good at writing. I got stressed, wondering if my ideas would be accepted, and I experienced anxiety about not being able to contribute.” (P14)

“I was hesitant to express my ideas because my peer insisted on having control over the decisions. Whenever I proposed an idea, he rejected it and insisted that his own idea was better, writing it instead.” (P17)

Another factor contributing to the emotional discomfort was the other party rejecting their ideas according to several participants (*n* = 6). P16 said:

“My peer found my sentences too simple and did not want to include them in the essay, which made me upset.”

With regard to interpersonal relationship dynamics, division of workload was not fair according to several participants (*n* = 9) as they had to take more responsibilities which led to experiencing negative feelings. P8 reported:

“My peer had no understanding of planning or formatting, so I had to explain these aspects to her and felt an increased responsibility for the task, which made me uneasy.”

Finally, participants reported a lack of mutual benefit in the collaboration (*n* = 12). P2 reported assuming most of the workload during the writing task and expressed frustration about this situation, noting that working with a more knowledgeable peer might have fostered mutual learning and greater benefits for both partners.

For social dimension, the participants collaborated to accomplish the task. Several participants (*n* = 7) exchanged ideas with the pair related to the essay topic. In addition, five participants reported creating a common piece from different thoughts. Collaborative writing supported for L2 writing skills improvement through peer assisted L2 learning (*n* = 3) and learning about L2 writing from the peer (*n* = 3) as stated by P20:

“My partner assisted me in figuring out how to write the essay.”

On the other hand, the participants faced obstacles while working together on writing. Difficulty of finding common ground (*n* = 15) was the major challenge according to the participants, which led to a waste of time for some of them (*n* = 4). P6 and P15 said:

“Both sides had different opinions, and the need to find common ground forced us to research our views further to make them more persuasive to the other party, as there was mutual distrust.” (P6)

“My peer was not open to criticism and acted as if he knew best. Therefore, it was difficult to find common ground.” (P15)

Trying to convince the other party that what they knew or wrote are wrong was another obstacle according to the participants (*n* = 5). P4 reported as follows:

“When I told my peer that her sentence or idea was incorrect, she would not be convinced, and I had to make extra effort to persuade her. She clearly did not know, but she still did not want to accept what I was saying.”

According to vast majority of the participants (*n* = 17), writing was more suitable to do individually as stated by the participants:

“I think I express myself better when writing alone. Whether good or bad, it fully belongs to me. But when we write together and receive feedback from the teacher, we start analyzing which parts have more mistakes based on who wrote them, and it makes us feel stressed.” (P2)

P2 prefers to take full responsibility for her writing, and this preference is influenced by the teacher's feedback, which makes her more aware of mistakes and adds pressure in a collaborative setting. Similar concerns were indicated by P5 and P8:

“When I write alone, all the responsibility belongs to me, but in collaborative writing, I feel uncomfortable when one side takes on less responsibility while the other takes on more. When we receive negative feedback from the teacher due to the other person's contribution, I feel upset and think, ‘This wouldn't have happened if I had written alone.' Accepting our own mistakes is easier.” (P5)

“I prefer writing alone because if there is a mistake, it is mine, and I want to learn from my own mistakes.” (P8)

Finally, P1 highlights the chaotic nature of collaboration:

“…it is like a single body being controlled by two heads. I love writing individually. Otherwise, you have to struggle with ideas that contradict your own and work to convince the other party to adopt your perspective.” (P1)

## 5 Discussion and conclusion

The purpose of this study was to investigate English language learners' engagement in collaborative writing by comparing student-selected and teacher-assigned pairings. The findings from both quantitative and qualitative data revealed systematic differences across student-selected and teacher-assigned high–low pairing configurations in the pair-level manifestations of social engagement (i.e., patterns of interaction) and cognitive engagement (i.e., focus of collaborative dialogue including LREs), as well as in participants' individual-level experiences of cognitive, affective, and social engagement. Overall, the findings indicate that different pairing configurations were associated with distinct dimensions of learner engagement. Student-selected pairs appeared to promote collaboration and positive engagement, whereas teacher-assigned high–low pairs showed advantages in some aspects of language-related problem solving. This may help explain inconsistencies in previous research on pairing arrangements.

As revealed in quantitative data, student-selected pairs displayed higher levels of social engagement, as evidenced by the prevalence of collaborative interaction patterns, while in teacher-assigned high–low pairs, the dominant/passive pattern was commonly observed, with the expert-novice pattern also emerging, though less frequently. Similarly, Storch and Aldosari (2013) found that high–low pairs adopted dominant-passive and expert-novice interaction patterns more than the collaborative pattern. On the other hand, these results contradict the findings of the study conducted by Basterrechea and Gallardo-del-Puerto (2020) with young EFL learners, which found that student-selected pairs adopted fewer collaborative patterns and showed more dominant-dominant and dominant-passive interaction patterns.

From the perspective of cognitive engagement, teacher-assigned high–low pairs devoted less attention to language-related problem solving. This finding aligns with recent evidence suggesting that mixed-proficiency configurations may reduce learners' observable cognitive engagement compared to same proficiency dyads (Wang et al., 2026). In contrast, the study conducted by Mozaffari (2017) revealed that teacher-assigned pairs (with similar levels) produced more LREs than student-selected pairs. In the present study, the teacher paired lower proficiency students with higher proficiency students, and these pairs adopted both dominant-passive and collaborative interaction patterns. One possible explanation is that in systematically structured high–low pairs, lower-proficiency students might have felt discouraged from challenging the ideas of their higher-proficiency partners and were more likely to accept their suggestions without question (Zabihi and Ghahramanzadeh, 2022). The interaction patterns observed in these pairs may partly account for the discrepancy between the two studies. Additionally, the nature of high–low pairing may have contributed to this difference, as Storch and Aldosari (2013) reported that high–low pairs generated more LREs when they adopted either the collaborative or expert-novice interaction pattern.

A significant difference was found in the number of unresolved or incorrectly resolved LREs, with student-selected (core participants with lower proficiency level) pairs generating more unresolved or incorrectly resolved LREs than teacher-assigned high–low pairs. This suggests that although friendship-based pairing may foster a comfortable interactional environment, it does not necessarily guarantee the successful resolution of language-related episodes when both partners possess limited linguistic resources. This finding is consistent with previous studies suggesting that proficiency composition plays an important role in successful LRE resolution, as mixed-proficiency pairs often achieve more successful collaborative dialogue outcomes than homogeneous low-proficiency pairs (e.g., Storch and Aldosari, 2013).

In teacher-assigned pairs, there was more focus on content rather than form, compared to student-selected pairings. This finding contradicts Wang (2024), who found that students engaged in more content-focused talk than form-focused talk when working in self-selected groups. Differences in peer familiarity between the two pairing configurations may have contributed to this pattern. In reflections and interviews, students reported that it was more difficult to find common ground when working with an unfamiliar peer. In contrast, they found it easier to accept and engage with their partner's ideas when working in student-selected pairs. These findings are consistent with those of the study by Putzeys et al. (2023) as they reported that negotiation was easier and tolerance was higher among friends in student-selected pairs.

While the quantitative analyses captured engagement as it was enacted during interaction through patterns of interaction and collaborative dialogue, the qualitative findings provide insight into how learners experienced these interactions. Together, these results revealed that observable interactional behavior and learners' subjective experiences were closely related but not always identical. Cognitively, peer learning was reported more frequently in student-selected configurations, whereas it was described less often in teacher-assigned high–low pairings. This is in line with recent findings indicating that learners' perceived social positioning within the dyad can strongly shape their cognitive engagement during collaborative tasks (Cao et al., 2026). Moreover, this finding aligns with Zabihi and Ghahramanzadeh (2022), who reported that higher-proficiency students were often unwilling to assist partners with lower proficiency. In teacher-assigned pairs, participants—especially those with higher proficiency—reported a lack of mutual benefit and an unequal division of workload which was also acknowledged in the study by Alawaji (2020).

As for affective dimension, positive emotions were reported more frequently in student-selected configurations than in teacher-assigned high–low pairings, a finding supported by Tsoi and Aubrey (2023). In this study, participants reported feeling free to express their opinions in student-selected pairs. This finding is consistent with Basterrechea and Gallardo-del-Puerto (2020), who found that young learners were more comfortable when working with a self-selected partner. In teacher assigned pairs, higher-proficiency students in particular complained about feeling obliged to take more responsibility and reported willingness to work with a more knowledgeable peer. Similarly, Zabihi and Ghahramanzadeh (2022) found that high-proficiency learners could become disappointed due to their low-proficiency partners' limited capacity to contribute effectively to the writing task. Moreover, this finding is consistent with the study by Putzeys et al. (2023), who found that students seek for mutual benefit in collaborative writing. Emotional discomfort arising from interpersonal exchanges were common among lower-proficiency students in teacher-assigned pairs, as they hesitated to express their ideas and often experienced rejection from higher-proficiency partners. This finding corresponds with Zabihi and Ghahramanzadeh's (2022) conclusion that lower-proficiency participants are likely to feel uncomfortable and inferior when working with higher-proficiency peers, which lowers their affective engagement.

Regarding social engagement at the individual level, participants in both sessions reported difficulty finding common ground; however, this challenge was more evident in teacher-assigned high–low pairs leading participants to conclude that writing is more suitable to be done individually. The main concern related to their preferences was about teacher feedback. Similarly, Zhai (2021) found that participants in her study preferred to be assessed individually, as they were concerned about receiving lower grades. Moreover, collaboration in task completion was prioritized in student-selected pairs in the study, likely due to the good relationships among friends. Since they were already comfortable with one another, they were less concerned about interpersonal issues, which likely enhanced the benefits of collaborative work (Wang, 2024).

The engagement patterns identified in this study can be interpreted from a social-constructivist perspective, which conceptualizes learning as a process facilitated through interaction and scaffolding (Vygotsky, 1978). The findings indicate that in addition to proficiency differences, affective and relational dynamics within pairs shape interaction in collaborative writing. Student-selected pairs, involving familiarity and more balanced proficiency, appeared to promote negotiation, reciprocal scaffolding, and shared responsibility. In contrast, teacher-assigned high–low pairs often led to unequal participation due to imbalanced scaffolding and affective discomfort that constrained open negotiation. Taken together, these findings suggest that differences in pairing configurations were associated with distinct affective conditions that shaped interaction patterns and related to variations in cognitive engagement, highlighting that cognitive, social, and affective engagement are interrelated rather than independent. This interdependence has also been highlighted in recent multidimensional models of engagement in collaborative writing (Zhang, 2025; Cao et al., 2026).

All in all, most participants expressed a preference for individual writing, a result that differs from findings in several previous studies including those by Abahussain (2020), Alawaji (2020), and Shehadeh (2011). Participants' dissatisfaction with collaborative writing appeared more strongly in the teacher-assigned high–low configuration. This configuration systematically paired students with differing proficiency levels and likely lower levels of prior familiarity, conditions that may have intensified the challenges they encountered. Overall, this study contributes to collaborative writing research by showing that pairing configuration is associated not only with interactional processes but also with learners' cognitive, social, and affective experiences. Using a within-subjects design and multiple data sources, it provides a more comprehensive understanding of engagement across classroom pairing arrangements. Consistent with Tsoi and Aubrey (2023), the findings suggest that the way pairing is structured in classroom contexts—including decisions regarding selection procedures and proficiency composition—may be an important factor for teachers to consider when designing collaborative writing tasks. More broadly, these findings suggest that pairing arrangements should be examined through multiple dimensions of engagement, rather than focusing solely on linguistic and interactional outcomes.

This study has some limitations. This study does not disentangle the effect of pairing method as pairing method was related to differences in proficiency configurations (homogeneous vs. high–low), presumably varying peer familiarity, and the use of different essay genres across conditions. Different essay genres (definition and comparison–contrast) were employed to reduce potential task repetition effects resulting from participants' repeated involvement in collaborative writing tasks. However, the genres may have differed in their rhetorical and cognitive demands, which could have influenced interactional patterns and engagement. Instead, two pedagogically meaningful classroom pairing configurations are examined in the study. Therefore, causal inferences about pairing strategy should be avoided. Each participant completed three collaborative writing tasks (one for familiarization, one in student-selected and one in teacher-assigned pairs). The short timeframe and limited number of collaborative writing tasks may not have allowed sufficient time for deeper engagement patterns to emerge or stabilize, especially in unfamiliar pairings. Future studies could employ a longitudinal design involving more writing sessions to track how engagement progresses over time. Furthermore, the study focused on engagement and interaction during specific writing tasks but did not explore long-term language development or writing improvement. Future studies could focus on long term effects of different pairing types. Finally, the effect of teacher feedback on learner engagement and collaboration dynamics could be explored in future studies, as participants in this study expressed concerns about teacher feedback, which likely influenced their preference for individual writing.

## Data Availability

The datasets presented in this article are not readily available due to ethical and privacy restrictions stipulated in the participant informed consent forms. Requests to access the datasets or anonymized excerpts should be directed to the corresponding author.
