# Supplementary material for: “….it is like a single body being controlled by two heads.”: exploring cognitive, social, and affective engagement in L2 collaborative writing
Source: Front Psychol. 2026 Jul 3;17:1849413. doi: 10.3389/fpsyg.2026.1849413 (PMC13382965; doi:10.3389/fpsyg.2026.1849413)
Supplement: Supplementary file 1 [file Table_1.docx]

**Coding for patterns of interaction:** Three patterns of interaction including collaborative, dominant/passive and expert/novice were observed in teacher assigned pair talks. In student-selected pairs the three patterns mentioned above were observed in addition to dominant/dominant pattern of interaction. The dialogues below use pseudonyms (only initial letters of pseudonyms are shown):

**Excerpt 1 (Collaborative):**

B:….face to face friendships..

İ:Why do not we use relationships instead of friendships?

B: ok we can write online relationships are common nowadays...

İ: In fact, face-to face friendships are common

B: we could write more common

İ: ok but would not it be better to write still more common?

B: yes, that would be better. Then, we could write face to face friendships or relationships are still more common…

In this excerpt, İ, more successful participant and B, less successful participant work together through consulting each other to complete the introductory paragraph.

**Excerpt 2 (Dominant/Passive):**

K:….nowadays we have online friendships and face-to-face friendships(he is writing what he is saying at the same time).

F: both of them have advantages and disadvantages

K: no, following is better:both of them have ups and downs

F:okay

K: with both having ups and downs, this is better.

F: okay, but to me..

K:(interrupting) with both having ups and downs (repeating). One can find one of them more reliable, but under what conditions?

F: then, we cannot take a side

K: I know what we are expected to do. I will complete the introductory paragraph: both of them…

F:okay.

In this excerpt, K, more successful and dominant participant, is the one who is writing the essay on the computer and deciding what to write most of the time. F, less successful one, tries to contribute to the writing but he is rejected or interrupted. He has misunderstanding about writing a compare-contrast essay (he thinks that they should have a position). K does not explain why he is wrong. He goes on writing as he knows.

**Excerpt 3 (Expert/novice):**

K: (starting to 1^st^ body) firstly,

İ: why do not we write initially instead of firstly?

K: ok, I am writing but does not it mean “lastly”?

İ: no, it means firstly, first of all. Since we use them frequently (first, first of all), we had better use a different linking word: initially.

K: ok, it sounds more academic. I have learnt something new. Thanks!

In this excerpt, İ, more successful participant, suggests using a different linking word which is new for K, less successful participant. He is happy for learning a new connector.

**Excerpt 4 (Dominant/Dominant):**

I:….makes your life better.

F: better? I think it should be ‘easier’, it sounds more academic.

I:in fact, better is more suitable.

F: but easier sounds more academic.

I: I think better is better!

Both participants insist on their word choices in this excerpt.

**Coding for LREs (Language related episodes):**

**Excerpt 1 (Form-Focused):**

M: …..online education differ from traditional education in that..

A: (interrupting)no, online education is singular and takes “s” so it should be “differs” not differ.

M: ahh, okay. (form-focused-solved)

**Excerpt 2 (Lexis-Focused):**

K:…sincerity is the milestone of friendship… but “milestone” sounds strange here, can we use another word instead of milestone?

F: we can use “an important matter of friendship” instead of “milestone”.

K: yes, it sounds better. (Lexis-Focused-solved)

**Excerpt 3 (Mechanics-Focused):**

M: …students can work together so they can socialize (the other participant is writing what he is saying) but you should not put a full stop; you should insert a comma before so.

A: ahh, okay. (mechanics-focused-solved)

**Excerpt 4 (Incorrectly resolved)**

Z: However, in online friendships…
S: You cannot know who your friend really is.
Z: Exactly.
S: We could also say: “You cannot know who your friend really are.”
Z: I do not think that is grammatically correct.
S: Do you mean the “you cannot know” part?
Z: Let’s use “in online friendships, you may not know” here: “You may not know who is your friends.”
S: Okay. *(Writes down what Z says.)*

In this excerpt, S initially produced the target-like form, *You cannot know who your friend really is*. However, the learners subsequently questioned its grammaticality and replaced it with the non-target-like form *You may not know who is your friends*. Because the learners reached an agreement and incorporated the incorrect sentence into their text, it was considered as incorrectly resolved.

**Excerpt 5 (Unsolved)**

İ: As in Walt Disney’s example…
F: Should we write, “As in Walt Disney’s example”?
İ: Yes, let’s add ’s there.
F: What does adding ’s mean?
İ: Let’s say, “As in Walt Disney’s example.”
F: It is not “Walt Disney’s example”; it is “the Walt Disney example.”
İ: The other one—the first one—makes more sense to me.
F: *(Repeating)* “The Walt Disney example,” rather than “Walt Disney’s example,” sounds better and more like a fixed expression.
İ: Anyway…

In this excerpt, the participants discussed whether to use the possessive construction *Walt Disney’s example* or the noun-modifier construction *the Walt Disney example*, but they did not agree on final solution. The discussion ended with “Anyway…,” indicating that the issue was abandoned.

**Content and Organization related talks**

**Excerpt 1 (Content related talk)**

Z: First, let’s mention that psychological violence is one of the things that harms society the most.
A: We can discuss its causes.
Z: Let’s identify the factors first.
A: We can proceed by giving examples. For instance, people who grow up in loveless environments and do not receive affection may be more inclined to engage in psychological violence.
Z: Exactly. Let’s also discuss juvenile offenders and their reasons for committing crimes.

In this excerpt, the participants generated, selected, and elaborated ideas to include in the essay, such as the causes of psychological violence and the effects of growing up without affection. The primary focus was on what to write even though the participants briefly talked about the order in which these ideas could be written.

**Excerpt 2 (Organization related talk)**

H: Let’s provide a definition in the introductory paragraph.
E: We can write the dictionary definition of love.
H: We can mention the subcategories of love in this paragraph.
E: Should we start with a question?
H: We can start with a question and then explain our own opinion.
E: What will our own definition be?
H: We could also begin with an interesting fact.
E: In the body paragraph, let’s also discuss the origin of the word “love.”
H: Okay.

In this excerpt, the dominant focus is on how to structure the essay, including what to place in the introduction, how to begin the text, and which information to include in the body paragraph although there is some content-related talk. Therefore, it was coded as organization related talk.
